# Impact of Multileaf Collimator Width and Normal Tissue Objective on Radiation Dose Distribution in Stereotactic Radiosurgery Using HyperArc for Single Brain Lesions

**DOI:** 10.3390/curroncol32050272

**Published:** 2025-05-07

**Authors:** Se An Oh, Jae Won Park, Ji Woon Yea, Jaehyeon Park, Yoon Young Jo

**Affiliations:** 1 Department of Radiation Oncology, Yeungnam University Medical Center, Daegu 42415, Republic of Korea; kapicap@ynu.ac.kr (J.W.P.); yjw1160@ynu.ac.kr (J.W.Y.); drjhyeon@ynu.ac.kr (J.P.); eurisko24@yu.ac.kr (Y.Y.J.); 2 Department of Radiation Oncology, Yeungnam University College of Medicine, Daegu 42415, Republic of Korea

**Keywords:** brain metastasis, HyperArc, stereotactic radiosurgery, normal tissue objective (NTO), multileaf collimator (MLC) width

## Abstract

This study retrospectively investigated the impact of stereotactic radiosurgery (SRS) normal tissue objective (NTO) and multileaf collimator (MLC) width on radiation dose distribution in patients with brain metastasis treated using HyperArc. In total, 21 patients who underwent SRS using the HyperArc of the TrueBeam linear accelerator from November 2022 to June 2024 were included. All patients received radiotherapy with HA_SH_ planned with SRS NTO and HD MLC. HyperArc(HA_AH_) combined with the auto NTO and HD MLC and HyperArc(HA_AM_) with auto NTO and millennium MLC were generated and compared. Monitor units (MU), conformity index (CI), radical dose homogeneity index (rDHI), moderate DHI (mDHI), and gradient index (GI) were evaluated as target factors, and V_2_(Gy), V_10_(Gy), V_12_(Gy), V_18_(Gy), V_10_(cc), and V_12_(cc) were evaluated as normal brain factors. Dosimetric comparisons were performed between HA_SH_, HA_AH_, and HA_AM_ and between target and normal brain tissues. Between HA_SH_ and HA_AH_, average MU was 7206 and 5798, respectively; the difference was significant (*p* < 0.001). The MU of HA_AM_ was 5835. Among HA_SH_, HA_AH_, and HA_AM_, CI and mDHI were not significantly different, but there were significant differences in rDHI, GI, and normal brain tissues. When treating a single lesion using HyperArc, SRS NTO influences MU and GI, and the MLC width influences rDHI and GI. In HyperArc for single metastatic brain lesions, SRS NTO and MLC width have a significant effect on the radiation dose delivered to the target and normal brain tissues.

## 1. Introduction

Brain metastases are becoming prevalent as advancements in cancer diagnostics and treatment have improved survival rates, resulting in increased cancer incidence. Brain metastases may result from various cancers and manifest with significant symptoms due to their potential to cause neurological complications [1,2,3]. The incidence of primary brain tumors is 6.6 per 100,000, while that for metastatic brain tumors is 8.3–11 per 100,000. The increasing prevalence of metastatic brain tumors is attributed to the prolonged survival of patients following the diagnosis of primary cancers [4].

Metastatic brain tumors are the most common malignant tumors in the cranial cavity and affect 9–15% of patients with cancer. Most cases of brain metastases are treated with a combination of surgical resection, chemotherapy, and radiotherapy [5]. Recently, HyperArc^TM^ (Varian Medical Systems, Palo Alto, CA, USA) has been used during stereotactic radiosurgery (SRS) to treat intracranial tumors [6,7,8,9,10,11,12].

Several institutions employ intensity modulation radiotherapy to deliver the desired radiation dose to tumors while minimizing radiation exposure to normal structures [13,14,15]. For an optimal radiation dose distribution, a multileaf collimator (MLC) is used to create complex movements over time. The millennium MLC has a 5 mm leaf width at the isocenter and is commonly used for standard IMRT and VMAT treatments. Conversely, the HD MLC offers enhanced resolution with 2.5 mm wide leaves in the central 8 cm region, improving the ability to conform doses for small or irregularly shaped targets. The finer leaf width of the high-definition (HD) MLC also allows for more accurate sparing of nearby critical structures while maintaining sharp dose gradients.

Ohira et al. investigated the effects of the MLC width on dose distribution for the treatment of multiple brain metastases using HyperArc and showed that high-definition (HD) MLC results in significantly better conformity, a steeper dose gradient, and greater normal tissue sparing than millennium MLC [16].

The most notable feature of the HyperArc plan is the SRS normal tissue objective (SRS NTO). SRS NTO, which can only be used with HyperArc, limits radiation exposure of normal tissues near target lesions. Additionally, SRS NTO is automatically selected in HyperArc and helps prevent unintended dose bridging between targets by limiting the dose between them to <17% of the prescription dose [6,8,9,17]. In contrast, in a conventional IMRT plan, the Automatic NTO (auto NTO) is usually selected by default. In IMRT planning, the auto NTO function automatically guides a smooth dose fall-off outside the planning target volume (PTV), minimizing high-dose spillage into normal tissues. Furthermore, it improves normal tissue sparing without requiring manual objectives and enhances planning efficiency and consistency. Auto NTO also stabilizes optimization by reducing the risk of hotspots and coldspots around the target.

There are several reports on the dosimetric characteristics of HyperArc using SRS NTO, its efficacy in cases such as benign brain lesions [6], comparisons between HyperArc and VMAT planning in single and multiple brain metastases [8], the use of HyperArc and multicriteria optimization in skull base meningiomas [17], and knowledge-based planning models trained with HyperArc plans for brain metastases [9]. However, there are no reports on the effects of SRS NTO and MLC width when treating single metastatic brain lesions.

Therefore, this study retrospectively investigated the effects of SRS NTO and MLC width on the radiation dose distribution in patients with single brain metastasis treated using HyperArc.

## 2. Materials and Methods

### 2.1. Ethics Statement

This retrospective study was approved by the Institutional Review Board (IRB) of Yeungnam University Medical Center (YUMC 2024-07-051). The requirement for informed consent was waived by the IRB as patient anonymity was ensured. Data access for research purposes commenced after IRB approval. We reviewed the medical records of patients with single brain lesions treated with SRS using HyperArc from November 2022 to June 2024.

### 2.2. Patient Selection

This study included 21 consecutive patients who underwent SRS using HyperArc from November 2022 to June 2024. Included patients were those with primary and metastatic brain tumors who underwent treatment of a single metastatic brain lesion. All patients were treated with SRS techniques using HyperArc technology and HD MLC. The characteristics of the patients and radiation treatments are presented in Table 1. All lesions received a dose of 20 Gy with 1 fraction. The volume of normal brain tissues was obtained by subtracting the gross target volume from the total brain volume; the average normal brain volume was approximately 1443.8 cc.

### 2.3. Immobilization and Computed Tomography (CT) Simulations

Patients were immobilized using the immobilization encompass™ SRS immobilization system (Avondale, PA, USA). According to the Yeungnam University Medical Center’s operation guidelines, the Brilliance Big Bore CT simulator (Philis Inc., Cleveland, OH, USA) was replaced by the Aquilion Exceed LB (Canon Medical System, Otawara, Japan) starting April 2024. All CT slices had a width of 1 mm. Thermoplastics for the SRS adapter were used with the SRS cushion support, including the MOLDCARE U head cushion.

**Figure 1:**
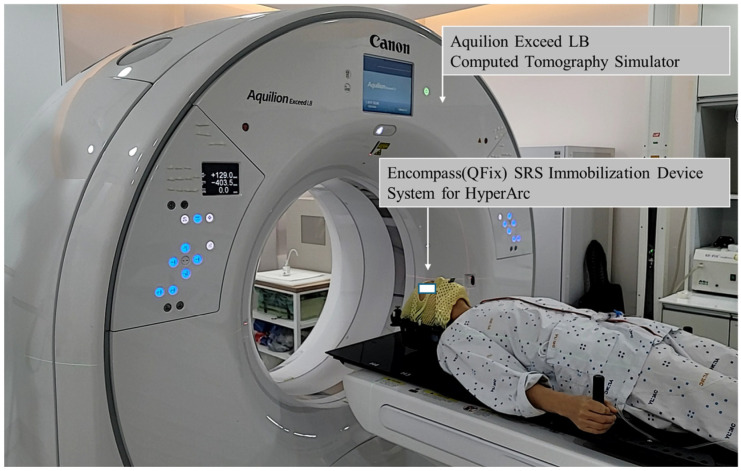
Simulation setup using the encompass SRS immobilization device system for HyperArc treatment on an Aquilion Exceed LB simulation CT scan.

Figure 1 shows the CT simulation setup with the QFix encompass^TM^ SRS immobilization system (Avondale, PA, USA) for radiotherapy using HyperArc.

### 2.4. Target Delineation

To improve the accuracy of contour delineation, a sequence of 2 mm axial and gadolinium-enhanced T1-weighted magnetic resonance images were fused to reference CT images. The gross target volume (GTV) was contoured by an experienced radiation oncologist and neurosurgeon with the assistance of the T1 and CT images. In 19 cases, the PTV was delineated at 1 mm from the GTV, while it was similar to the GTV in 2 cases.

### 2.5. Treatment Planning

Overall, 21 consecutive patients with one metastatic brain lesion underwent SRS NTO and HD MLC (HA_SH_). Using the HA_SH_ treatment plan, we aimed to evaluate the effects of SRS NTO and MLC width on radiation dose distribution. We also created HA_AH_, which is a treatment plan for auto NTO and HD MLC, to further compare the effects of SRS NTO on radiation dose distribution. Additionally, we also created HA_AM_ treatment plans using auto NTO and millennium MLC to evaluate the effects of the MLC width on radiation dose distribution.

HyperArc optimizes the collimator angle and field size to minimize the radiation doses delivered to normal tissues according to the relationship between the normal and target organs. Furthermore, the beam geometry of HyperArc automatically arranges a four-arc field; one of the arc fields creates a coplanar full or half field at a couch angle of 0°, and the other three arc fields form non-coplanar half arcs at couch angles of 45°, 90° (or 270°), and 315° [8].

All radiation treatment plans were created in Eclipse ARIA 15.6 Versions (Varian Medical System, Palo Alto, CA, USA), and the calculation algorithm employed was the Acuros XB advanced dose calculation algorithm (AXB, Varian Medical System, Palo Alto, CA, USA). Generally, gamma knife plans prescribe the dose to the 50% isodose line, whereas that for linac-based systems is 80–100%. Moreover, linac-based systems offer flexibility in dose normalization methods, allowing for different approaches to optimize dose distribution within the target. In our study, instead of prescribing to a specific isodose line, we employed a normalization method wherein 100% of the prescription dose was delivered to the minimum dose within the PTV. Additionally, normal tissue tolerance was optimized to satisfy the TG-101 guidelines [18].

The reference HA_SH_ treatment plans set dose constraints to deliver the prescribed dose to the target lesions while minimizing the radiation exposure of normal tissues. HA_AH_ and HA_AM_ treatment plans were also optimized with the same settings as those of HA_SH_.

### 2.6. Comparative Dosimetric Evaluation of Target and Normal Brain Tissues

Radiation doses to the target and normal brain tissues between the HA_SH_, HA_AH_, and HA_AM_ plans were compared using the dose volume histogram (DVH). Additionally, the conformity index (CI) was used to quantitatively evaluate the target coverage. Van’t Riet et al. [19] calculated the CI as follows:

(1) $$Conformity\;Index\left(CI\right)=\frac{{TV}_{RI}}{TV}\times \frac{{TV}_{RI}}{V_{RI}}$$

Here, TV_RI_ is the TV covered by the reference isodose and V_RI_ is the volume of the reference isodose. In this study, the reference isodose was set at 100% of the prescribed dose.

To analyze the uniformity of dose distribution to the target tissues, the homogeneity index (HI) was assessed. Oliver et al. [20] proposed the radical dose HI (rDHI) and moderate DHI (mDHI), which are less affected by steep dose gradients near the boundary or small hotspots that are calculated as follows:

(2) $$radical\;Dose\;Homogeneity\;Index\left(rDHI\right)=\frac{D_{min}}{D_{max}}$$

where D_min_ is the minimum dose to the PTV and D_max_ is the maximum dose to the PTV.

(3) $$moderate\;Dose\;Homogeneity\;Index(mDHI)=\frac{D_{\;\geq 95\%}}{{D\;}_{\geq 5\%}}$$

where D_≥95%_ is the dose to 95% of the volume of the PTV and D_≥5%_ is the dose to 5% of the volume of the PTV.

Furthermore, we calculated the gradient index (GI), which is a quantitative indicator that characterizes the decrease in radiation dose with increasing distance from the outer surface of the TV. Park et al. [21] calculated the GI as follows for the dosimetric evaluation of HyperArc™ and RapidArc™ plans:

(4) $$Gradient\;Index\left(GI\right)=\frac{V_{prescription\;50\%}}{V_{prescription\;dose}}$$

Here, V_prescription dose_ is the volume receiving the prescription dose and V_prescription dose 50%_ is the volume receiving 50% of the prescription dose.

A major side effect of SRS is the radiation necrosis of adjacent brain tissues, which occurs in 5–26% of cases after approximately 6–11 months [22]. Radiation necrosis, which causes neurological changes, may require treatment with steroids, surgery, bevacizumab, and hyperbaric oxygen therapy [23], and several studies have reported on its risk and predictive strategies [24,25]. In this study, the volume delivered by each radiation dose was analyzed using V_2Gy_ (%), V_10Gy_ (%), V_12Gy_ (%), V_18Gy_ (%), V_10Gy_ (cc), and V_12Gy_ (cc).

### 2.7. Statistical Analysis of HA_SH_, HA_AH_, and HA_AM_ Plans

This study analyzed the data of 21 patients who underwent SRS NTO and HD MLC using HyperArc. Owing to the nonparametric nature of the data, the Wilcoxon signed-rank test was used to compare differences between plans using SRS NTO and conventional auto NTO as well as between HD MLC and millennium MLC. All statistical analyses were conducted using IBM SPSS Statistics (Version 29 SPSS, Chicago, IL, USA), with *p*-values < 0.05 considered statistically significant.

## 3. Results

**Figure 2:**
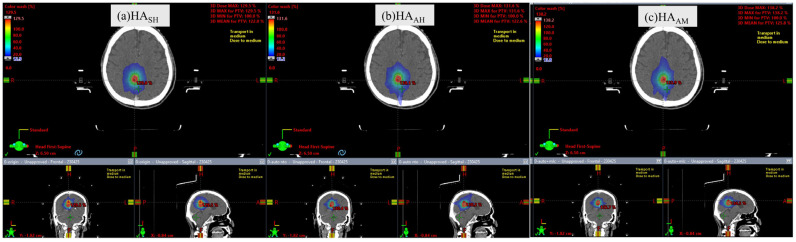
Comparison of radiation dose distribution of HA_SH_, HA_AH_, and HA_AM_ plans in transversal, frontal, and sagittal planes in patient #7.

Figure 2 shows the comparison of radiation dose distributions with the HA_AH_, HA_AH_, and HA_AM_ plans in the transverse, frontal, and sagittal planes in patient #7. Dose distributions between the HA_SH_, HA_AH_, and HA_AM_ were not significantly different.

**Figure 3:**
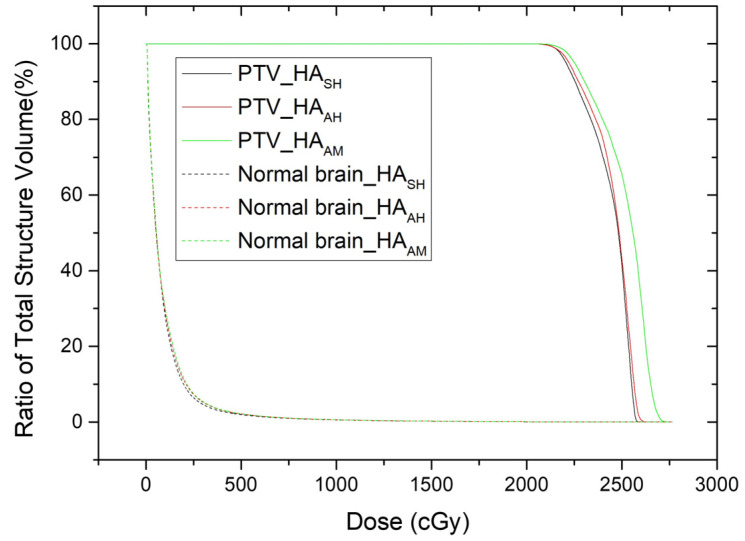
Comparison of dose volume histogram of HA_SH_, HA_AH_, and HA_AM_ plans for PTV and normal brain tissue in patient #7.

Figure 3 shows the DVH of the HA_SH_, HA_AH_, and HA_AM_ plans for the PTV and normal brain tissue in patient #7. Doses delivered to normal brain tissues were similar in all treatment plans, while the maximum dose to the PTV was similar for HA_SH_ and HA_AH_ but not for HA_AM_, which was significantly higher. A Wilcoxon signed-rank test was performed to quantitatively examine the differences.

The HA_AH_, HA_AH_ and HA_AM_ plans were compared using the monitor units (MU), Max of PTV, Mean of PTV, CI, rDHI, mDHI, and GI for the PTV, and V_2_(Gy), V_10_(Gy), V_12_(Gy), V_18_(Gy), V_10_(cc), and V_12_(cc) for normal brain tissues, as summarized in Table 2.

### 3.1. Plan Comparison of HA_SH_ and HA_AH_

To verify the efficacy of SRS NTO, the HA_AH_ and HA_AH_ plans were compared. The MU of HA_SH_ was 7206.37 ± 1102.81, while that of HA_AH_ was 5798.0 ± 1511.99, showing a significant difference (*p* < 0.001). Meanwhile, the CIs of HA_SH_ and HA_AH_ were 0.49 ± 0.19 and 0.50 ± 0.20, respectively, showing no significant difference (*p* = 0.784). The GIs of HA_SH_ and HA_AH_ were 3.87 ± 1.44 and 5.52 ± 2.27, respectively, showing a significant difference (*p* < 0.001). Additionally, the doses delivered to normal brain tissues were significantly different between HA_SH_ and HA_AH_ (*p* < 0.05).

### 3.2. Plan Comparison of HA_AH_ and HA_AM_

The HA_AH_ and HA_AM_ plans were compared to determine the doses delivered between millennium MLC and HD MLC according to the MLC width. The MUs of HA_AH_ and HA_AM_ were 5798.04 ± 1511.99 and 5835.41 ± 1536.52, respectively, showing no significant difference (*p* = 0.434). The CIs of HA_AH_ and HA_AM_ were 0.50 ± 0.20 and 0.47 ± 0.19, showing no significant difference (*p* = 0.083). The GIs of HA_AH_ and HA_AM_ were 5.52 ± 2.27 and 5.90 ± 2.78, respectively, showing a significant difference (*p* < 0.033). In terms of V_2_(Gy), V_10_(Gy), V_12_(Gy), V_18_(Gy), V_10_(cc), and V_12_(cc), the doses delivered to normal brain tissues between HA_AH_ and HA_AM_ were significantly different (*p* < 0.05).

## 4. Discussion

Twenty-one patients with single brain lesions were treated with the SRS techniques in one fraction at a dose of 20 Gy using HyperArc with SRS NTO and HD MLC (HA_SH_). To investigate the influence of SRS NTO and MLC width on the treatment plan for HyperArc with a single lesion, we generated HAAH plans using auto NTO and HD MLC and HAAM plans using auto NTO and millennium MLC.

Regarding SRS NTO, Ho et al. [6] compared HyperArc, CyberKnife (CK), and RapidArc (RA) plans in fractionated stereotactic radiotherapy for 16 benign brain lesions. HyperArc plans using SRS NTO resulted in significantly lower V_5Gy_, V_12Gy_, V_24Gy_, and mean brainstem dose than RA plans. Additionally, the HyperArc plans showed better dose distribution with a lower radiation exposure of normal tissues than the CK plans. SRS NTO automatically generates a virtual shell around the target lesion and performs rapid dose reduction to prevent dose bridging to adjacent targets.

Ohira et al. [8] compared the dosimetric parameters of conventional VMAT and HyperArc plans in 23 patients with 1–4 brain metastases. They revealed that the HyperArc plan with SRS NTO for single and multiple targets showed high conformity and rapid dose fall-off compared to conventional VMAT, and the radiation necrosis predictive factor (V_8Gy_–V_16Gy_) was significantly reduced. However, our findings revealed that SRS NTO did not show a significant difference, and other factors showed similar results to those of Ohira et al. Compared to the study by Ohira et al. [8], which evaluated HyperArc planning for single and multiple brain metastases, our study focused exclusively on single brain lesions and specifically investigated the individual effects of SRS NTO implementation and MLC width. While both studies demonstrated improvements in CI and dose gradient, differences were noted in MU, GI, and dose homogeneity. These may be attributed to variations in patient cohort characteristics, planning objectives, and technical parameters. Notably, using SRS-specific NTO optimization and HD MLCs in our study may have contributed to further MU reduction and improved GI compared with the results of Ohira et al.

This study assessed the impact of the MLC width during HD MLC and millennium MLC on the radiation dose distribution when there is one lesion in the brain with HyperArc.

Ohira et al. [16] conducted a study on 21 patients with 5–10 metastatic brain lesions who underwent fractionated SRS using HyperArc that employed either HD MLC and millenium MLC. Their findings revealed that the CI of HD MLC (0.95 ± 0.04) was significantly higher than that of millennium MLC (0.92 ± 0.06) (*p* < 0.0001). Additionally, the GI of HD MLC (5.6 ± 2.5) was significantly lower than that of millennium MLC (6.2 ± 3.5) (*p* < 0.0001). Furthermore, the radiation dose delivered to normal brain tissues in HD MLC was significantly lower than in millennium MLC in all dose ranges (V_6Gy_–V_28Gy_) (*p* < 0.0001). However, in our study, HD MLC was not significantly different from millennium MLC according to the CI, whereas other factors had similar results. The difference in results may be attributed to the number of targets affecting the CI when treating brain tumors with HyperArc-based SRS. Compared to Ohira et al. [16], who studied the MLC width in HyperArc fSRT for multiple brain metastases, our study focused on single-session SRS for single lesions. While both studies showed improved GI with HD MLCs, differences were noted in MU and dose homogeneity. The use of SRS NTO, which was not employed by Ohira et al., and the differences in lesion number and fractionation likely contributed to the variations in dosimetric outcomes.

This study has several limitations. First, the total number of patients was only 21, which seems insufficient; more meaningful results would have been obtained if the number of patients was higher. Second, HA_SH_ and HA_AH_ were generated by the same machine, whereas HA_AM_ was generated by a different machine. Although linear accelerators underwent regular quality inspections and were maintained within clinical tolerances, slight differences in beam modeling, mechanical tolerances, and machine characteristics may have introduced minor uncertainties in dose calculation and delivery parameters. Nevertheless, the similarity in MU between HA_AH_ and HA_AM_ indicates that machine-related variabilities only had minimal impacts on the comparative results. Moreover, all treatment plans were created using the same version of the Eclipse treatment planning system and standardized planning protocols, minimizing potential inconsistencies related to system configuration.

## 5. Conclusions

SRS NTO, compared to auto NTO, showed a significant increase in MU. Although the CI and GI significantly improved, the doses delivered to normal brain tissues were significantly reduced (*p* < 0.05). In addition, HD MLC significantly reduced the GI and the dose delivered to normal brain tissues while maintaining an almost similar MU as millennium MLC (*p* < 0.05). Therefore, when using HyperArc for single metastatic brain lesions, we recommend using both SRS NTO and HD MLC.

## Figures and Tables

**Table 1:** Characteristics of the patients and radiation treatments in this study.

Patient Characteristics	
Number of patients	N = 21
Median age (range)	66 (47–85)
Gender (%)	
Female	6 (28.6)
Male	15 (71.4)
Radiation Treatment Characteristics	
Dose of prescriptions (%)	
20 Gy	21 (100)
Number of fractions (%)	
1	21 (100)
Size of volume (cc)	
GTV	1.18 (0.03–12.00)
Planning target volume (PTV)	2.04 (0.10–21.20)
Normal brain (cc)	1443.8 (1190.8–1679.8)

GTV = gross target volume; PTV = planning target volume; Normal brain (cc) = brain (cc)–GTV (cc).

**Table 2:** Parameters for target volumes and normal brain tissues with HA_SH_, HA_AH_, and HA_AM_ in brain stereotactic radiosurgery plans.

	Parameters	HA_SH_	HA_AH_	HA_AM_	*p*-Value	
		Mean ± Standard Deviation	Mean ± Standard Deviation	Mean ± Standard Deviation	HA_SH_ vs. HA_AH_	HA_AH_ vs. HA_AM_
Target	MU	7206.37 ± 1102.81	5798.04 ± 1511.99	5835.41 ± 1536.52	<0.001 *	0.434
	Max of PTV	125.70 ± 5.21	122.39 ± 5.23	124.38 ± 6.11	0.010 *	0.033 *
	Mean of PTV	116.18 ± 4.50	113.37 ± 4.69	114.53 ± 5.17	0.003 *	0.032 *
	CI	0.49 ± 0.19	0.50 ± 0.20	0.47 ± 0.19	0.784	0.083
	rDHI	0.80 ± 0.03	0.82 ± 0.04	0.81 ± 0.04	0.010 *	0.046 *
	mDHI	0.86 ± 0.06	0.88 ± 0.03	0.88 ± 0.02	0.848	0.122
	GI	3.87 ± 1.44	5.52 ± 2.27	5.90 ± 2.78	<0.001 *	0.033 *
Normal brain	V_2Gy_ (%)	3.43 ± 4.81	4.27 ± 4.55	4.64 ± 4.26	0.001 *	0.006 *
	V_10Gy_ (%)	0.25 ± 0.27	0.33 ± 0.29	0.38 ± 0.32	<0.001 *	<0.001 *
	V_12Gy_ (%)	0.18 ± 0.19	0.22 ± 0.20	0.26 ± 0.22	<0.001 *	<0.001 *
	V_18Gy_ (%)	0.05 ± 0.05	0.06 ± 0.06	0.07 ± 0.07	<0.001 *	<0.001 *
	V_10Gy_ (cc)	3.63 ± 4.21	4.74 ± 4.44	5.47 ± 4.91	<0.001 *	<0.001 *
	V_12Gy_ (cc)	2.57 ± 2.88	3.26 ± 3.02	3.82 ± 3.46	<0.001 *	<0.001 *

HA_SH_ = HyperArc combined with the SRS NTO and high-definition (HD) MLC; HA_AH_ = HyperArc combined with the auto NTO and HD MLC; HA_AM_ = HyperArc combined with the auto NTO and millennium MLC; MU = monitor units; CI = conformity index; rDHI = radical dose homogeneity index; mDHI = moderate dose HI; GI = gradient index; V_nGy_ = volume receiving at least nGy; * *p* < 0.05.

## Data Availability

The original contributions presented in this study are included in the article. Further inquiries can be directed to the corresponding author.

## Abbreviations

The following abbreviations are used in this manuscript:

CI	Conformity index
CK	CyberKnife
CT	Computed tomography
DVH	Dose volume histogram
GI	Gradient index
GTV	Gross target volume
HI	Homogeneity index
IMRT	Intensity-modulated radiotherapy
IRB	Institutional Review Board
MLC	Multileaf collimator
MU	Monitor units
NTO	Normal tissue objective
PTV	Planning target volume
SRS	Stereotactic radiosurgery
VMAT	Volumetric modulated arc therapy
